# Extraction of Alkalis from Silicate Materials Part 1—Amorphous Silicate Materials

**DOI:** 10.3390/ma15124056

**Published:** 2022-06-07

**Authors:** Wiktor Szewczenko

**Affiliations:** Faculty of Civil Engineering, Mechanics and Petrochemistry, Warsaw University of Technology, Łukasiewicza Str. 17, 09-400 Płock, Poland; wiktor.szewczenko@pw.edu.pl; Tel.: +48-243672239

**Keywords:** glass, extraction, alkalis, alkaline activity, kinetic, diffusion

## Abstract

The main building materials widely used worldwide are those based on cement, glass, and ceramics. Taking into account the fact that the raw material base for the production of these materials is narrowing, and the quality of raw materials is declining, methods are being used to modify the structure of silicate materials in order to improve their properties when using cheaper raw materials and industrial waste, which should help reduce the energy intensity of their production. One of the ways to reduce energy consumption is the use of alkaline components in the chemical composition of silicate materials, which makes it possible to reduce the temperature of their synthesis. However, the presence of alkalis in the material at the stage of the operation is undesirable since it contributes, for example, to a decrease in the chemical resistance of silicate glasses or leads to the phenomenon of alkaline corrosion in cement products. In this regard, in order to reduce the negative impact of alkalis, it is necessary to extract them from the surface layers of the silicate material. There are various methods for extracting alkalis from silicate materials, some of which are presented in this article.

## 1. Introduction

Silicate materials can exist in nature in amorphous and crystalline states, for example, as inorganic glass and cements, which allows us to call these materials antagonistic. They are characterized by a similar chemical composition but different quantitative contents (Table 1).

The introduction of alkali oxides into the composition of most industrial silicate glass is motivated due to the fact that alkali oxides, while forming eutectic compounds with silica, reduce the melting temperature of the molten glass. Alkalis in the composition of raw materials act similarly during the formation of clinker in the kiln during cement production. However, the presence of alkalis in glass and cements impairs their properties.

It is known that the alkaline component of neo-organic glass contributes to a decrease in its chemical resistance when impacted by an aggressive environment [9]. This is explained by the fact that alkaline cations in the composition of glass are modifiers that “loosen” its structure; therefore, an increase in the content of alkali oxides in glass contributes to the deterioration of its physical and chemical properties. Alkali oxides in the composition of glass increase its electrical conductivity, thereby reducing its electrical insulating properties, and also increase its coefficient of thermal expansion, thereby reducing heat resistance, etc.

When comparing the positive and negative effects of alkaline oxides in the composition of silicate glass, it can be concluded that they are extremely necessary to ensure technological properties, but their negative impact on the operational properties must be minimized. One of the ways to solve this problem is the controlled extraction of alkalis from glass.

**Figure 1:**
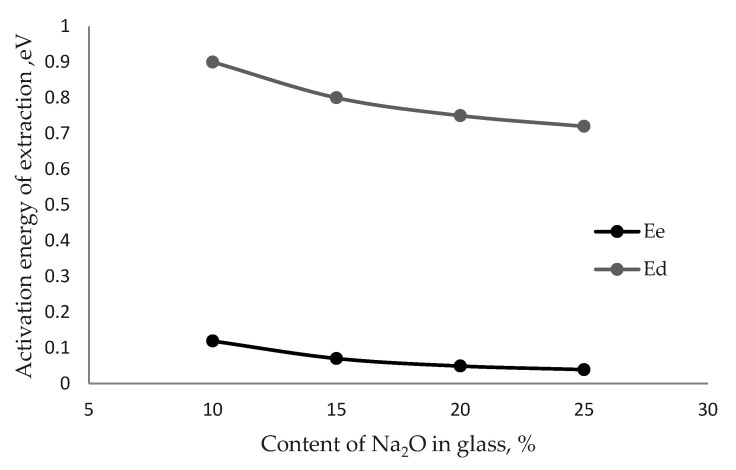
Dependence of the activation energy of extraction Ee and Ed on the content of Na_2_O in glass.

Alkaline cations in materials can be in various energy states. For the same value of the ionic bond energy, the energy state of the cation will depend on its environment. Surface cations have a low activation energy during extraction due to their non-equilibrium state. At the same time, alkali cations located in the deep layers have higher activation energies compared to the surface ones. Thus, the process of extraction of alkali cations consists of two components: (a) kinetic, in which only alkali surface cations participate and which is described by the activation energy Ek, and (b) diffusion, which is described by the activation energy Ed. Compliance with the condition Ek < Ed was confirmed in [10], using the example of model glasses (Figure 1).

In the example of two-component glasses of the Na_2_O–CaO–SiO_2_ system, it was shown that Ed is 14 times higher than Ek. This is due to the fact that the alkaline component of silicate glasses is presented in the structure in the form of modifier cations, which are ionically bonded to the silicate network. The value of the ionic bond energy of the Si-O^−^ Na^+^ bond depends on the ionic radius of the cation and is always less than the energy of the covalent bond Si=O [11].

Until now, to quantify the alkali content of a glass surface, traditional methods for determining chemical resistance were used, based on the long-term boiling of glass powders or shaped glass surfaces in water, followed by titration of a 0.01 N solution with a solution of hydrochloric acid [12].

Thus, the quantitative assessment of the energy state of alkali cations on the glass surface during short-term contacts with the extractant during the extraction process can be a characteristic of the chemical state of the glass surface. This characteristic is essentially a new property of glass, which we have called alkaline activity [13].

In contrast to traditional methods for determining chemical stability, the method for determining the alkaline activity of glass is based on a short contact time (up to 30 s) of a glass object (exposure time) with an extractant, followed by quantitative determination of the content of alkaline catiogens in the solution by flame photometry [14].

In addition, it should be noted that the energy state of alkaline cations is influenced by their environment. For example, the activation energy of surface alkali cations is much lower compared to that of cations in the bulk of the glass. This is explained by the fact that the surface is a part of a system that is in contact with the external environment and, therefore, at least one side is not affected by the system; this is the reason for the lower binding energies of surface cations. In general, it looks like a condensed system (glass) is in an equilibrium state until contact with the external environment (non-condensed system) due to the action of certain factors beginning to interact with each other. External factors should be understood as the effects of various chemical reagents (water, acids, alkalis, and reactive gaseous reagents). During the extraction of alkaline cations from the glass surface, regardless of the type of the extractant, vacancies remain on the glass surface, the negative charge of which must be instantly compensated for by the positive charge of the cation from the surface layers of the glass to maintain the equilibrium state of the surface. Thus, during the extraction, a diffusion mechanism occurs of one-way motion of alkali cations from deep layers to the surface. Alkaline cations extracted from glass, depending on the type of extractant, can form the corresponding hydroxides (during low-temperature extraction) or salts (during high-temperature extraction).

Inorganic glasses are characterized by two types of alkaline extraction:

1—High temperature, in the temperature range close to the temperature of the beginning of plastic deformation (softening) of the majority of silicate glasses of industrial composition (500–600 °C);

2—Low temperature, in the temperature range up to 100 °C.

The first of these can be conditionally divided into thermal and thermochemical. The difference between them is that, during thermal extraction, the extraction products are removed from the glass, while during thermochemical extraction, the reaction products between the alkaline component of the glass and the extractant are formed on the glass surface, regardless of its type.

For a quantitative assessment of the alkaline extraction process, a method of gas-flame treatment of the glass surface was developed. The essence of the method is that the investigated glass surface is subjected to gas-flame treatment at a high heating rate (20–40 °C/S). At the same time, using a photometer, the temperature at which the surface glasses begin to release alkaline cations is recorded, as evidenced by the coloration of the flame. The criterion for assessing the alkaline activity is the temperature at the beginning of this extraction process (Tbe).

**Figure 2:**
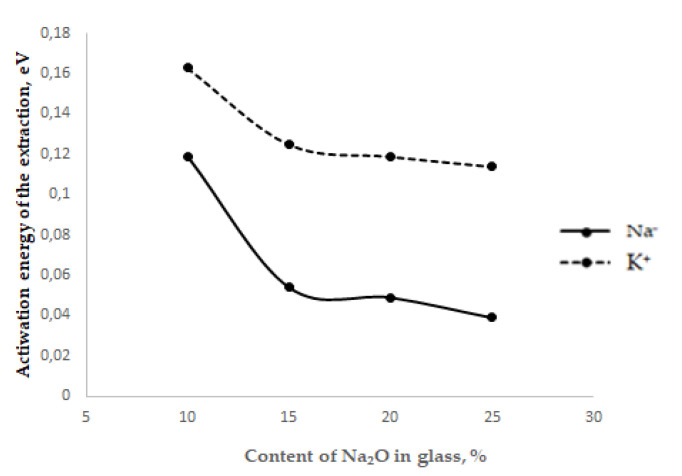
Dependence of the activation energy of the extraction process on the type and content of alkalis.

The results of studying the alkaline activity of two- and three-component glasses of the Na_2_O–CaO–SiO_2_ system showed that an increase in the content of various alkaline oxides in glass is accompanied by an increase in alkaline activity, and a decrease in the activation energy of the extraction process (Figure 2).

Comparing the activation energy of the extraction process (Ee) of surface alkali cations with the diffusion activation energy (Ed) for the same glass compositions, it can be noted that Ee is almost two orders of magnitude lower than Ed. This difference indicates a nonequilibrium state of alkali metal cations on the glass surface [15].

In two-alkali glasses with a total sodium oxide and potassium oxide content of 20%, with an increase in the content of sodium oxide from 5 to 15%, there is a decrease in Tbe for Na^+^ by 50 °C and an increase in Tbe for K^+^ by 154 °C. For K^+^, the same picture is seen in the example of an increase in extraction activation energy [10].

In [16], the glass surface was treated with a weakly ionized plasma, and the alkaline activity of the glass surface was determined by the change in the plasma electrical conductivity. By the graphical dependence of the extraction process intensity on the temperature in Arrhenius coordinates, the activation energy of the extraction process was determined, which ranged within 41.8–83.6 kJ/mole for window glass.

These data indicate that potassium cations are much more strongly bound on the glass surface than sodium ions when they are present together in the glass composition. The temperature difference at the start of extraction (Tbe) for sodium and potassium ions means that during mandatory heat treatment (annealing) of glass containers, the ratio of alkalis on the glass surface is disturbed, which leads to a partial loss of the poly-alkaline effect, designed to increase the chemical resistance of the glass containers.

Considering the fact that the alkaline activity of Portland cement, as a representative crystalline silicate system, is limited by the relevant standards, the addition of powdered glass waste to the cement leads to an excess alkali content of the cement–glass mixture [17].

In this regard, any uncontrolled changes to the alkali content of the glass can lead to uncontrolled changes to the alkali content of the cement–glass mixture, which can affect, for example, the durability of concrete.

Thus, when using industrial glass waste as an additive to Portland cement, it is necessary to maintain control over the alkaline activity of the glass waste used.

Various methods have been proposed for controlling the alkaline activity of glass based on various types of alkaline extraction.

**Figure 3:**
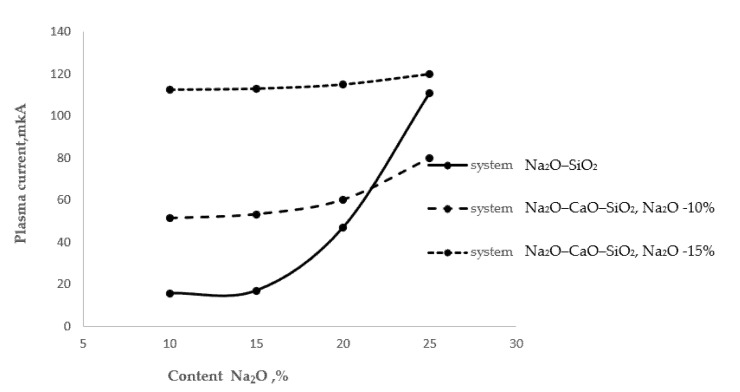
High-temperature extraction of alkali cations from various glasses.

To control the alkaline activity of the glass surface, the phenomenon of high-temperature extraction can be used. As is known, in the high-temperature zone of a gas flame, a partially ionized gaseous medium is formed—a low-temperature isothermal plasma [16]. At a stable flow rate of gas and air at a constant ratio, plasma is characterized by a certain degree of ionization α, and its electrical conductivity is a certain value of the magnitude of the current, Ipl. In the process of heating the glass surface with a gas flame, at a certain temperature—let us call it the temperature of the beginning of the extraction process, Tne—alkaline ions begin to evolve from the glass. When they enter the plasma flow, they sharply increase the electrical conductivity of the plasma. Thus, by controlling the temperature on the glass surface and simultaneously measuring the magnitude of the plasma current, it is possible to determine this temperature Tne. Figure 3 shows the process of extraction of alkaline cations from glasses with different surface alkali contents.

As follows from Figure 3, as the alkali oxide content in the two-component glass of the Na_2_O–SiO_2_ system increased, the thermal extraction also increased. A particularly sharp increase in the amount of extracted sodium cations was observed in the glass with a 25% Na_2_O content. In the three-component glass of the Na_2_O–CaO–SiO_2_ system, at a stable Na_2_O content of 10% and with an increase in the CaO content from 10 to 20%, the plasma current increased by 17%, which may be associated with the extraction of sodium cations. In the same three-component glass with a stable Na_2_O content of 15%, the plasma current increased by 2.2%. An almost twofold increase in the plasma current should be noted in comparison with the 10% Na_2_O content glass, probably associated with the extraction of calcium cations.

**Figure 4:**
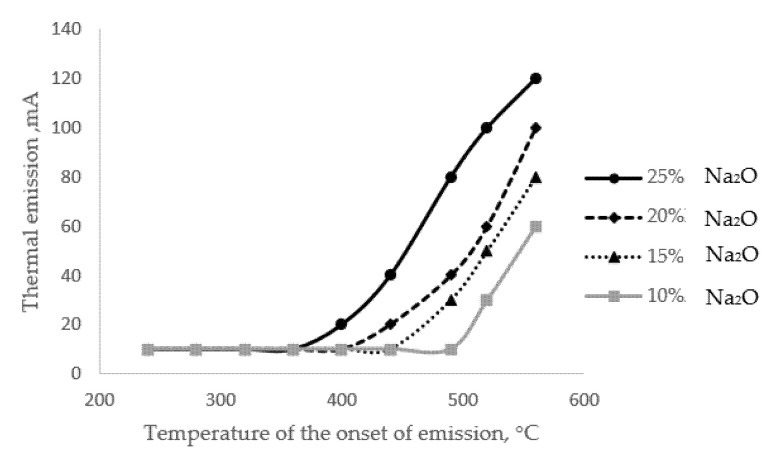
Activated thermal emission curves for glasses with different alkali contents.

In addition to the method described above for determining the alkaline activity of a glass surface, one can use the method of activated thermionic emission by measuring the electric potential of a medium saturated with alkali cations [18]. As mentioned above, the temperature of the onset of emission (extraction, tne) primarily depends on the alkali content of the glass (Figure 4).

The results presented in Figure 4 show that there is a close relationship between the alkali content of the glass and the initial temperature of the extraction process; the correlation coefficient for this relationship is 0.9. The phenomenon of thermal emission itself is a special case of the alkaline extraction process, which has a diffusion character.

The methods of high-temperature alkaline extraction also include thermochemical extraction [19,20,21].

It is known [9] that the presence of sulfur dioxide in the atmosphere surrounding glassware at temperatures close to the maximum upper annealing temperature leads to the interaction of the gas with the alkaline component of glass according to the scheme:

SO_2_ + 2Na^+^ + O_2_ → Na_2_SO_4_

gas glass air glass surface

The amount of sodium sulfate formed depends on the temperature of the thermochemical treatment and its duration. With increasing temperature, the amount of sodium sulfate increases with a stable processing time. However, the amount of sodium sulfate formed on the glass surface at a constant temperature and duration will depend only on the alkali content on the glass surface. It should be noted that the mode of thermochemical treatment depends on the type of glass; for example, for window and container glass, it is 550 °C with a duration of 0.5–1 min [22].

If we assume that the amount of extracted alkali is directly proportional to the concentration of alkali metal cations per unit volume of the surface layer of finite thickness, and the coefficient of diffusion of alkali metal cations through this layer, then the amount of extracted alkali can be calculated by using the formula:

Q = 4Co^2^·D/(π √ Ʈ)

where: Q is the amount of alkali extracted (mg/cm^2^), Co is the concentration of sodium ions per unit volume of the surface layer of glass, D is the diffusion coefficient, and Ʈ is the duration of the thermochemical treatment (min).

In the case of thermochemical extraction, the duration of the extraction process is almost 25 times higher than that for the standard method for determining water resistance, which can be explained by the higher diffusion coefficient [23].

However, approximately the same amount of extracted alkalis Q [24], at the level of 0.4–0.5 mg Na_2_O/dm^2^ in low-temperature ion-exchange and high-temperature thermochemical extraction methods, can be explained by different mechanisms of the extraction process. In [20], the authors found that the process of high-temperature extraction occurs in the kinetic region. This means that the overall rate of the extraction process is not limited by diffusion, as in the case of ion-exchange extraction, but by the rate of reaction between sulfur dioxide and the alkaline component of the glass surface. In this case, the factor limiting the overall rate of the extraction process is the deposition of reaction products on the glass surface. Thus, it can be assumed that the shorter the duration of the contact of the extractant with the glass surface, the better the amount of extracted cations will reflect their initial concentration on the surface. This condition is best met by the method for determining thermal extraction, according to which the duration of the extraction process is two orders of magnitude less than that in thermochemical methods and three orders of magnitude higher than that in ion-exchange methods (Table 2).

The extraction process, which is based on ion exchange, is the basis of the traditional method for determining the water resistance of glass [9]. Upon contact with water, an ion exchange reaction takes place:

Si-O^−+^Na + H^+^OH^−^ → Si-O^−^ H^+^ + NaOH

Glass water glass solution

As a result of the action of water on the glass, its surface is hydrated, and the extractant turns from neutral to alkaline.

**Figure 5:**
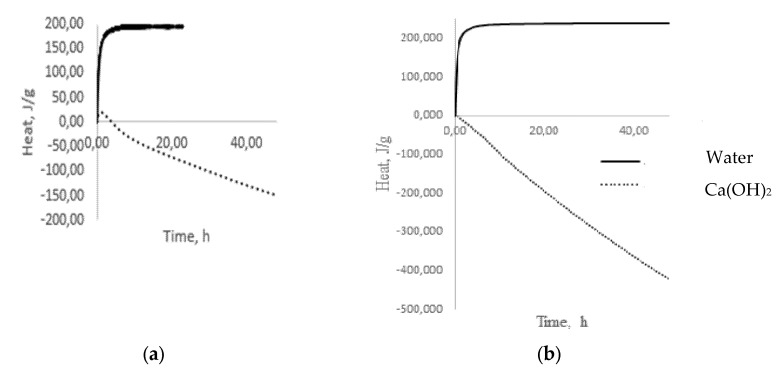
Dependence of heat release on time when glass comes into contact with water and Ca(OH)_2_: (**a**) green glass; (**b**) low-alkali borosilicate glass.

As is known, water in contact with glass at the initial stage acts selectively as a reagent of the first group, extracting only alkaline cations from glass [12]. The latter, with OH^−^ groups of water, forms alkalis, which already belong to the reagents of the second group, completely dissolving glass. The results of microcalorimetric studies testify to the formation of alkalis (Figure 5a) [25]. The process of alkali formation proceeds most actively in the first 60 min from the moment of glass coming into contact with water, as evidenced by intense heat release (Figure 5a), after which the stabilization period begins, indicating the completion of the selective extraction stage and the transition to the glass dissolution stage, accompanied by heat absorption (Figure 5b).

A completely different picture emerges when glass is exposed to, for example, Ca(OH)_2_, which belongs to the reagents of the second group, which dissolve glass. During the first hour, there is a slight exo-effect (Figure 5), which can be associated with the formation of intermediate products and which decreases to zero over the next three hours. Then, the process of heat absorption is observed, which can be identified as a dissolution process (Figure 5). A similar picture forms in the case of low-alkali borosilicate glass (Figure 5), with the only difference being that there is no exo-effect in the interaction with Ca(OH)_2_ in contrast to multi-alkali container glass. With prolonged contact of the glass with water at temperatures up to 100 °C, the concentration of the alkaline solution increases, and it begins to act on the glass as a reagent of the second group that dissolves glass. In this case, the process of alkaline extraction can be conditionally divided into two stages: kinetic, in which only surface alkaline cations participate, and diffusion, in which alkaline cations from deeper layers of glass participate. The first of them is fast because of the low activation energies for the extraction of surface alkaline cations, and the second is slow because it is associated with diffusion. This separation of the extraction process into two stages is very important since, in some cases, it becomes necessary to assess the alkaline activity in short time intervals.

For example, when glass powder is used as an additive to Portland cement, alkaline cations of the first kinetic phase of extraction have a significant effect on the process of cement hydration in the pre-induction period of hydration; in parallel with alkaline extraction, the formation of hydrosilicate and calcium hydroxide occurs with a sharp increase in the pH of the medium [26].

Under such conditions, the use of traditional methods for determining the water resistance of glass to assess its extraction ability is impossible due to the transition of the extractant from a neutral state to alkali, which allows us to take part in the kinetic phase of the extraction process.

To assess the alkaline activity of the glass surface, we can use the change in the pH of water at normal temperature (20 °C) after the introduction of a portion of the glass. In this case, the amount of extracted ions will depend on the specific surface area of the glass powder [27].

Given the fact that, in accordance with the European standard EN 197-1, for cement composites in which part of the clinker cement is replaced by various hydraulic and pozzolanic additives, the factor of the total alkali content in such composites is of great importance, and the amount of alkali cannot exceed 2.0% [28].

Currently, there are many works are devoted to the issue of using glass waste as additives to Portland cement [7,29,30,31,32,33,34,35,36,37].

However, if we talk about the possibility of using glass waste in a powdered state as an additive to Portland cement, it is necessary to take into account the contribution of the alkaline component of the glass to the total alkalinity of cement composites [17]. This issue is discussed in detail in [27]. In this case, the ratio of the amount of alkaline ions on the glass surface to that in the volume is very important.

The extraction of alkali ions from the glass surface leads to the emergence of diffusion flows from deep layers due to the concentration gradient. It is extremely difficult to take into account their influence on alkaline activity; therefore, the minimum possible exposure time is a prerequisite for assessing the alkaline activity of powder materials. This approach makes it possible to calculate the alkaline activity from the alkali content of the glass. Until now, it was believed that the chemical stability of glass, as one of its few properties, could not be calculated. However, this becomes possible if we assume that the glass is chemically homogeneous and that the glass powder particles are cube-shaped. In this case, the ratio of the amount of alkali ions on the surface to their content in the volume will be approximately 0.6 for a cube face size of 1 mm^2^. In this case, the alkaline activity will be equal to [27]:

Ac = C·Ce·S^−1^

where Ac is the calculated value of the alkaline activity of the glass powder (mg Na_2_Oeq/m^2^), C is the total alkali content of the glass (mg Na_2_Oeq/g glass), Ce is the amount of extracted Na_2_Oeq (%), and S is the specific surface area of the glass powder (m^2^/g).

The calculated alkaline activity values for the most common container glasses are 4.12, 3.84, and 4.54 mg Na_2_Oeq mg/m^2^ for colorless, brown, and green glass, respectively [27].

The given data thus indicate that brown container glass is the most suitable for use as the constituent of Portland cement.

## 2. Conclusions

Based on the presented material, the following conclusions can be drawn:

- The process of alkaline extraction consists of two components: kinetic, in which surface alkaline cations take part, and diffusion, in which alkaline cations of the deep layers of the substance take part;
- The activation energy of the extraction process of alkaline cations depends on the amount of alkalis in the glass composition;
- Increasing the extraction temperature of silicate materials in the amorphous state increases the amount of the extracted substance;
- To assess the chemical state of the surface of silicate materials in the amorphous state, the concept of alkaline activity is proposed, which includes the amount of extracted alkaline cations from the glass surface with a short exposure time;
- Evaluation of the alkaline activity of powdered silicate materials in the amorphous state makes it possible to evaluate the contribution of glass additives to the alkaline activity of Portland cement, as a representative of silicate materials in the crystalline state.

## Figures and Tables

**Table 1:** Chemical compositions of antagonistic silicate materials.

Material	Oxides, wt %												References
	SiO_2_	Al_2_O_3_	Fe_2_O_3_	CaO	MgO	Na_2_O	K_2_O	SO_3_	Cr_2_O_3_	TiO_2_	BaO	B_2_O_3_	
Glass, colorless	72.17	1.35	0.07	10.92	1.10	13.13	0.61	0.09	<0.05	0.06	<0.05	-	[1]
	73.04	1.81	0.04	-	10.75	-	13.94	0.22	-	-	-	-	[2]
Glass, green	71.30	2.18	0.59	-	12.18	-	13.07	0.05	0.44	-	-	-	[3]
	72.38	1.49	0.29	11.26	0.54	13.52	0.27	0.07	0.13	0.04	-	-	[4]
Glass, brown	72.08	2.19	0.22	10.45	0.72	13.71	0.16	0.05	<0.05	0.10	<0.05	-	[1]
	71.19	2.38	0.29	10.38	1.70	13.16	0.70	0.04	-	0.15	-	-	[5]
Glass, borosilicate	81.0	2.00	-	0.5	-	4.50	-	-	-	-	-	12.00	[6]
CEM I 32.5R	20.33	4.65	3.04	61.78	3.29	0.24	0.59	3.63	-	-	-	-	[7]
CEM I 42.5R	20.20	4.70	3.00	61.9	2.60	0.19	0.82	3.90	-	-	-	-	[8]

**Table 2:** The dependence of the amount of extracted Na_2_O on the extraction method.

Extraction Method	Extractor	Extraction Temperature, °C	Extraction Time, min	Amount of Extracted Na_2_O, mg/dm^2^	References
thermal	natural gas	330–440	0.18	17.00	[16]
thermochemical	SO_2_	500–600	15	0.34	[16]
ion exchange	H_2_O	95–98	60 300 360	0.35 0.40 0.42	[21]

## Data Availability

The data presented in this study are available on request from the corresponding author.

## References

[1] Zhu H., Chen W., Zhou W., Byars E.A. (2009). Expansion behaviour of glass aggregates in different testing for alkali-silica reactivity. Mater. Struct..

[2] Park S.B., Lee B.C., Kim J.H. (2004). Studies on mechanical properties of concrete containing waste glass aggregate. Cem. Concr. Res..

[3] Park S.-B., Lee B.-C. (2004). Studies on expansion properties in mortar containing waste glass and fibers. Cem. Concr. Res..

[4] Shayan A., Xu A. (2004). Value-added utilisation of waste glass in concrete. Cem. Concr. Res..

[5] Sobolev K., Türker P., Soboleva S., Iscioglu G. (2007). Utilization of waste glass in ECO-cement: Strength properties and microstructural observations. Waste Manag..

[6] Kotsay G., Brzóska A. (2021). Effect of Borosilicate Glass Wastes and Synthetic Silica on Cement Products Properties. Chem. Chem. Technol..

[7] Shi C., Wu Y., Riefler C., Wang H. (2005). Characteristics and pozzolanic reactivity of glass powders. Cem. Concr. Res..

[8] Schwarz N., Cam H., Neithalath N. (2008). Influence of a fine glass powder on the durability characteristics of concrete and its comparison to fly ash. Cem. Concr. Compos..

[9] Dubrowo S. (1965). Glass for Laboratory Products and Chemical Equipment.

[10] Shevchenko V. (1987). Selective leashing of the surfacee of alkali silicate glasses during heat treatment. Glas. Phys. Chem..

[11] Handke M. (2008). Krystalochemia Krzemianów.

[12] Pavlushkin N., Sentyurin G., Khodakovskaya R. (1970). Practicum on the Technology of Glass and Glass-Ceramics.

[13] Roy S. (1977). The Chemical Physics of Surfaces.

[14] Poluektov N. (1959). Flame Photometry Analysis Methods.

[15] Hannay N.B. (1971). Solid-State Chemistry.

[16] Shevchenko V. (1988). Leaching of the glass surface during its treatment with a weakly ionized plasma. Phys. Chem. Glass.

[17] Szewczenko W., Kotsay G. (2021). Alkaline Activity of Portland Cement with Additives of Waste Glass. Materials.

[18] Shevchenko V., Kuchera Y.I. (2004). Control of the chemical state of the surface of sheet glass. Glass Ceram..

[19] Shevchenko V., Redko L. (1985). Leaching of sheet glass surface. Glass Ceram..

[20] Shevchenko V. (1986). Thermal leaching of the glass surface. Glass Ceram..

[21] Shevchenko V. (1988). Selective leaching of glass and methods of its control. Glass Ceram..

[22] Yashishin Y., Shevchenko V., Gorbay Z. (1974). Increasing the strength of sheet glass. Glass Ceram..

[23] Smets B., Lommen T. (1981). SIMS and XPS investigation of the leahing of the glasses. Verres Refract..

[24] Shevchenko V. (1999). Chemical Diagnostics and Surface Modification of Silicate Glass.

[25] Shevchenko V.V., Kotsay G.N. (2020). Influence of Glass Powder Additives on the Hydration Process of Portland Cement. Glas. Phys. Chem..

[26] Shevchenko V.V. (2012). ASR effect in glasses used as additives to Portland cement. Glass Phys. Chem..

[27] Shevchenko V.V., Kotsai G.N. (2016). Alkaline activity of glass powder used as additives to Portland cement. Part II. Glas. Phys. Chem..

[28] (2013). Cement—Part 1: Composition, Specifications and Conformity Criteria for Common Cements.

[29] Shayan A., Xu A. (2006). Performance of glass powder as a pozzolanic material in concrete: A field trial on concrete slabs. Cem. Concr. Res..

[30] Khmiri A., Chaabouni M., Samet B. (2013). Chemical behaviour of ground waste glass when used as partial cement replacement in mortars. Constr. Build. Mater..

[31] Jani Y., Hogland W. (2014). Waste glass in the production of cement and concrete—A review. J. Environ. Chem. Eng..

[32] Aliabdo A.A., Abd Elmoaty A.E.M., Aboshama A.Y. (2016). Utilization of waste glass powder in the production of cement and concrete. Constr. Build. Mater..

[33] Wang Y., Li J., He X., Zheng Z., Su Y., Zhao H., Yang J., Strnadel B. (2020). Effects of wet-grinded superfine waste glass on the fresh properties and reaction characteristic of cement pastes. Constr. Build. Mater..

[34] Liu G., Florea M.V.A., Brouwers H.J.H. (2019). Performance evaluation of sustainable high strength mortars incorporating high volume waste glass as binder. Constr. Build. Mater..

[35] Abdelli H.E., Mokrani L., Kennouche S., de Aguiar J.B. (2020). Utilization of waste glass in the improvement of concrete performance: A mini review. Waste Manag. Res. J. Sustain. Circ. Econ..

[36] Bueno E.T., Paris J.M., Clavier K.A., Spreadbury C., Ferraro C.C., Townsend T.G. (2020). A review of ground waste glass as a supplementary cementitious material: A focus on alkali-silica reaction. J. Clean. Prod..

[37] Serpa D., Santos Silva A., de Brito J., Pontes J., Soares D. (2013). ASR of mortars containing glass. Constr. Build. Mater..

